# Supplementary material for: Comprehensive Analysis of Granzymes and Perforin Family Genes in Multiple Cancers
Source: Biomedicines. 2025 Feb 7;13(2):408. doi: 10.3390/biomedicines13020408 (PMC11853441; doi:10.3390/biomedicines13020408)
Supplement: Supplementary file 1 [file biomedicines-13-00408-s001.zip › biomedicines-3313663-supplementary.pdf]

Supplementary Data

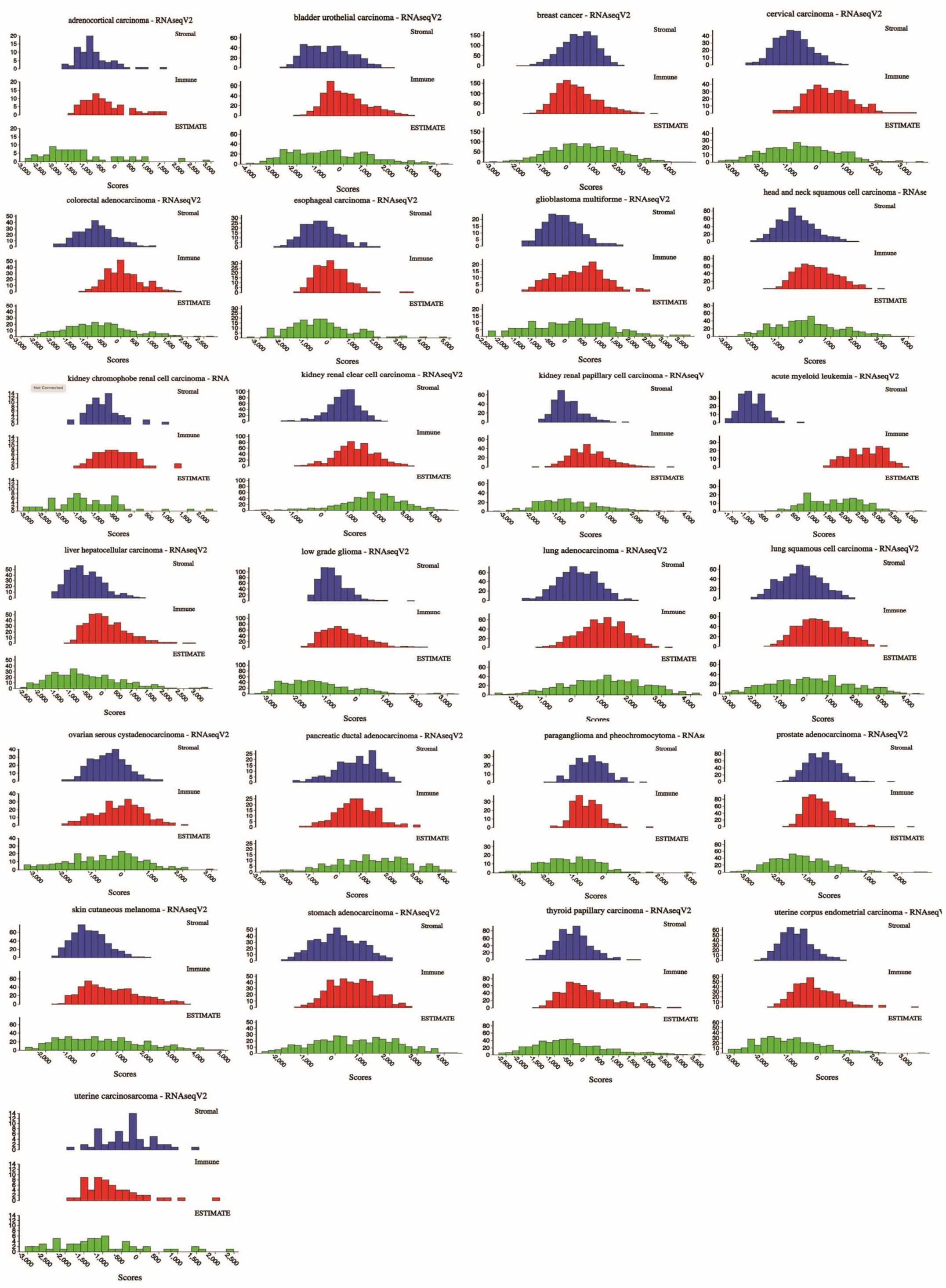

Figure S1: Stromal, Immune and ESTIMATE score ranges for different tumor samples.

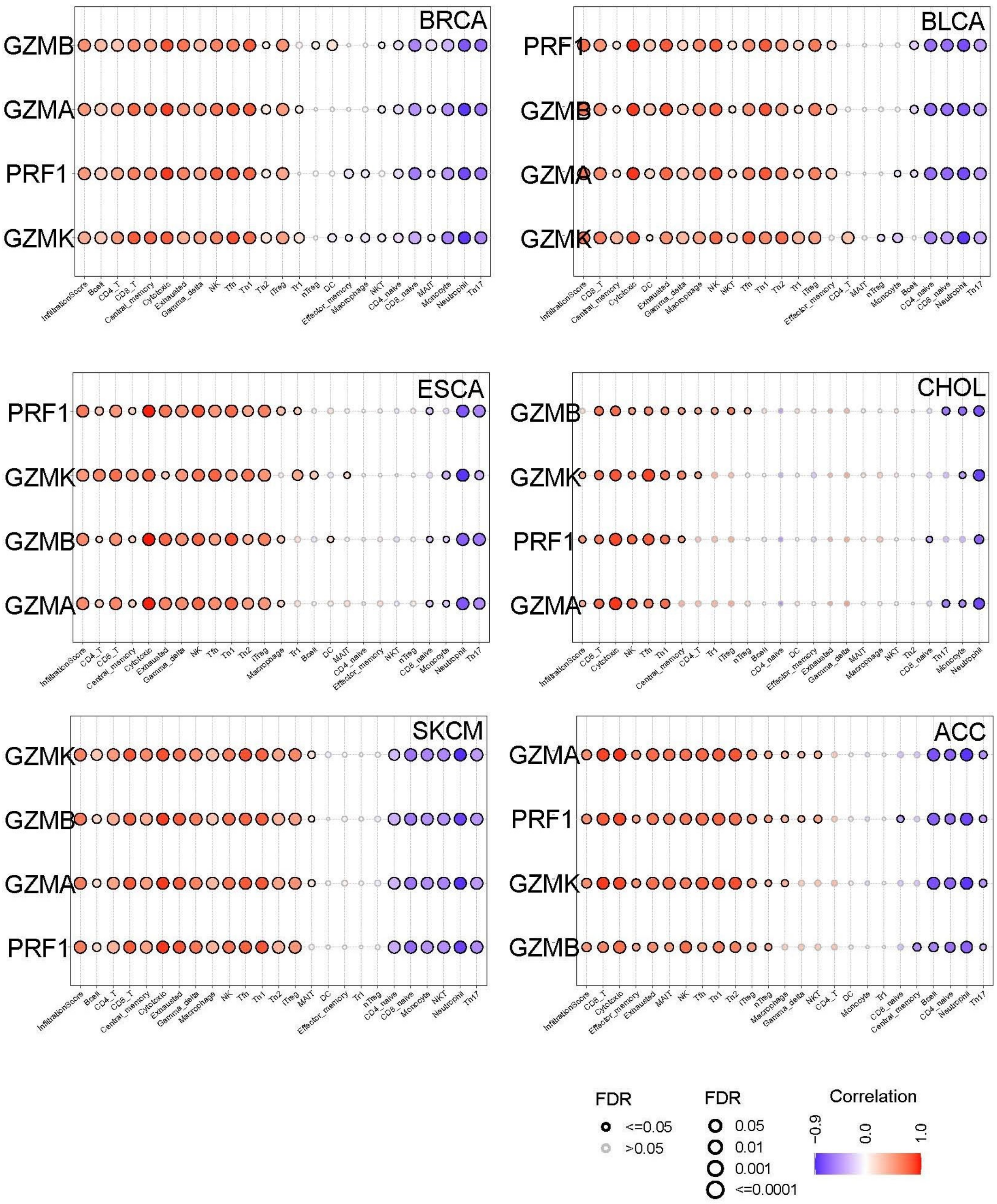

Figure S2. Bubble plots display correlation between gene expression (*GZMA*, *GZMB*, *GZMK* and *PRF1*) and immune infiltrates in BRCA, BLCA, SKCM, ESCA, CHOL and ACC cancers.
